# Inclusive electroencephalography practices for Black and Muslim participation in a White non-Muslim-majority country: A co-produced tutorial

**DOI:** 10.3758/s13428-026-03203-w

**Published:** 2026-10-14

**Authors:** Damian Cruse, Ilham Razaie, Nyamesumpa Abbey, Natasha Bradbury, Yasmin Darawil, Hana Eldessoky, Fatima Farooq, Olivia Francis, Romy Froemer, Dorinda Lokko, Esther Nwakor, Amaliah-Saffron Odutola, Andrew Quinn, Nishita Sujan, Malaika Zabair

**Affiliations:** 1 School of Psychology, University of Birmingham, Edgbaston, B15 2TT UK; 2 Centre for Human Brain Health, University of Birmingham, Edgbaston, B15 2TT UK

**Keywords:** Electroencephalography, Inclusivity, Black, hijab, Hair type

## Abstract

Most electroencephalography (EEG) research is conducted in White-majority Western countries by White researchers. EEG practices therefore often explicitly and implicitly exclude minoritised groups, leading to low diversity in EEG data samples that deepens biases in interpretation and clinical utility. Because EEG electrodes must contact the scalp, Black people and Muslim women who wear a hijab (or, Hijabis) are two systematically excluded groups. As a group of EEG researchers, Black people, and Hijabis, here we describe co-produced practical approaches to overcoming EEG participation barriers, from recruitment to data collection, and provide co-produced text, photographs, and videos designed to break down these barriers. These approaches provide further tools for the EEG community to tackle systemic exclusion and foster better, more representative neuroscience.

## Introduction

Countless studies of human psychology and multiple clinical contexts measure brain activity with electroencephalography (EEG; Mushtaq et al., 2024). However, because EEG requires electrodes to be attached to the participant’s scalp, many communities are directly and indirectly excluded from contributing to EEG samples, while White participants are significantly over-represented (Bradford et al., 2024; Choy et al., 2022; Penner et al., 2023; Ricard et al., 2023). This is a critical issue, as systemic exclusion from EEG research limits the generalisability of findings and ultimately reinforces distrust of medical science among marginalised groups (Corbie-Smith et al., 2002).

As the diversity of academic researchers does not reflect the diversity of the general population (Mba et al., 2024), ‘co-production’ provides a key means for researchers to ensure that their research best addresses the needs of underrepresented communities. Consequently, here we describe the outcomes of a co-production project (a collaborative research approach that brings together academics and community members) to identify and address barriers to EEG participation faced by two marginalised communities—Muslim university students who wear hijab, and Black university students. As most EEG participants are likely to be university students in White non-Muslim-majority countries (Henrich et al., 2010), the outcomes of our work have applications across the broad EEG research community. We hope that this manuscript serves as a tutorial for both more inclusive EEG methods and the co-production approaches that foster them.

## Direct and indirect exclusion of Black and Hijabi participants from EEG research

The hijab is a religious garment worn by Muslim women. Hijabis—women who wear a hijab —do not take off their hijabs in front of men. For Muslim women, the hijab is both an item of clothing and a lifestyle that reflects personal boundaries for physical contact with men, which in this context includes no touching of the head/hair as required by EEG. As a significant proportion of research EEGs are conducted by postgraduate students and postdocs, who are approximately 50% male (Machlovi et al., 2021), Hijabi women can contribute data to significantly fewer EEG studies than non-Hijabi women. Furthermore, exclusion from EEG research for Hijabis is a racialised experience, as only ~ 6% of British Muslims are White (Muslim Council of Britain, 2024) compared with ~ 80% of British academics and PhD students (Mba et al., 2024). While similar racialised experiences of exclusion are likely across all religious head-coverings, here we focus on Hijabi experiences due to the high representation of Muslim students within our potential participant pool and the additional racialised experiences of Hijabis in the United Kingdom.

A further racialised experience of exclusion from EEG research is felt by the Black community. EEG research systems typically house electrodes within a headcap, allowing somewhat consistent electrode placement. However, when participants have hair that is dense, curly, or coily, it is often impossible to fit the cap in such a way that allows good electrode connection to the scalp. As this hair type is typical with Black participants—sometimes known as afro-textured hair—EEG researchers may actively exclude Black participants in order to avoid signal quality challenges (also known as ‘phenotype bias’, Brown et al., 2023; Webb et al., 2022). Furthermore, differences in EEG signal quality between participants with different hair phenotypes may result in apparent, yet spurious, racial differences in brain activity, further feeding into harmful views of race as biological rather than socially constructed (Bradford et al., 2024).

The low ethnic diversity of Western academia means that most EEG researchers are ill-equipped to recognise, understand, and respond to these issues. While some guidelines and recommendations exist for more inclusive practices (e.g., https://hellobrainlab.com/research/eeg-hair-project/; https://sprweb.org/wp-content/uploads/2025/12/SPR-CPSI-DO-EEG-Hairtypes-Infographic-2025.pdf), EEG researchers often lack explicit training, which can lead to negative experiences for minoritised participants that further contributes to low engagement with EEG research (Louis et al., 2022). Alongside the imperative to remove the systemic barriers that limit the diversity of voices and experiences across levels of academic careers (Mba et al., 2024), co-production is a valuable method for bringing marginalised voices into research environments to ensure that research procedures meet the needs of all.

## A co-production approach to addressing exclusion from EEG research

Like many EEG researchers, our potential participant pool is composed primarily of university students. We therefore focused our work on students’ perspectives. To identify and begin to address barriers to EEG participation, we (DC, RF, AQ) advertised via departmental communications, lectures, and student societies for applicants to join paid working groups of six Hijabi undergraduate students and six Black undergraduate students. These working groups were to function as PPIE (patient and public involvement and engagement) groups, providing expertise from their lived experience to identify barriers and develop solutions. We selected our group members based on evidence in their applications that they valued inclusivity and worked with their communities. Each group completed a combination of independent work and group meetings, chaired by the first author (DC). To ensure that an unconstrained range of issues and solutions were considered, our working groups’ discussions were initially student-led. Over time, the chair guided task-oriented discussions around the foci identified by group consensus, bringing other researchers and operations staff into group meetings when valuable. When the groups had finalised their recommendations, they designed, scripted, storyboarded, and starred in two short videos per group with the aim of informing participants and researchers, respectively. As our goal was to provide resources designed for these communities by these communities, both the chair and the films’ production team (all White non-Muslim men) aimed for minimal editorial input.

To support our groups’ discussions, we also sought the views and experiences of our university’s student cohort via an online survey (ethical approval provided by the University of Birmingham STEM Ethics Board), advertised in lectures and via student societies, with each respondent entered into a draw to win one of twenty £10 online shopping vouchers. Alongside demographic questions, the survey was composed of a series of open questions about the respondents’ experiences of EEG research, any barriers to participation that they perceived, and what they felt would help to remove those barriers (see Appendix A for full survey). Note that we did not conduct formal qualitative analysis of the free-text responses, as the purpose of our survey was to provide our working groups with access to additional views from across our student cohort. Consequently, our working groups read the free-text responses and identified what they considered to be the key topics raised by respondents about barriers and potential solutions, and incorporated these into their co-production work.

Two hundred people contributed usable data to our survey, with ~ 19% reporting wearing a hijab and ~ 14% describing themselves as Black/African/Caribbean. Our survey’s quantitative data supported our anecdotal observations, with 48% of White respondents reporting having been participants in EEG research (57/109) compared with 21% (5/24) of Black respondents. While 54% of Hijabis (20/37) reported having participated in an EEG study compared with 22% of all participants who did not wear a religious head-covering (26/118), this distinction may be driven by sampling bias, as our recruitment targeted those with experiences of EEG research within this community.

From the free-text survey responses and our working group’s discussions, the unique perceived challenges and barriers for Hijabis revolved around the topic of ‘hesitancy’—not knowing whether the researcher would be a man or not knowing whether the hijab would ‘*get in the way*’ of them completing the study. One survey respondent noted that they were ‘*not sure if [they] could approach the researchers and ask if [they] were able to participate*’. Hijabi recommendations for increasing their EEG participation revolved around ‘accommodations’—‘*Having female researchers present, preferably if they were Muslim too, would increase participation in my demographic*’; ‘…*assurance that requesting a female researcher would not be an issue*’—and ‘transparency’—‘*A clear separate sheet of info on how they accommodate religious headwear so I can see if it’s suitable for me*’; ‘*Statements that say how they will be accommodating to participants and how this will be actioned*’.

From the free-text responses of our Black participants and our working group’s discussions, the unique perceived challenges and barriers revolved around the topic of ‘hair’—‘*Not having an appropriate hairstyle, some studies state … you must not have cornrows*’; ‘*I know they’d likely reject me because of my braids so I don’t bother trying*’—and ‘past experiences’—‘*After getting turned away one time, I assumed that all other experiments would have similar barriers and so I stopped signing up*’—with at least one survey respondent viewing this as explicitly racialised exclusion—‘*I was rejected because of my race*’. This group’s recommendations followed similar topics to our Hijabi respondents; specifically, ‘accommodations’— ‘*A new way of gathering information that allows thicker hair textures to participate*’—and ‘transparency’—‘*I think I would be more motivated if I was told in advance what actions the researcher was going to take to accommodate my hair. I would then participate if I felt these adjustments were genuine and comfortable*’.

## EEG Procedures: Recommendations by and for Hijabis

The fundamental barrier to participation in EEG research for Hijabis is the presence, or potential for presence, of a man when they will need to remove their hijab. In the absence of pre-existing guidance (Mushtaq & Ibáñez, 2025), we propose that removing this barrier requires a two-fold approach. First, male researchers must be able to request a female EEG ‘operator’ to collect data when their participant is a Hijabi and, second, the Hijabi participant must feel secure in the knowledge that, even with a female EEG researcher/operator, there is no possibility for a man to enter the room uninvited.

Consequently, we recommend that research centres employ a paid female EEG ‘operator’. Specifically, we co-produced the duties and processes of a female EEG operator role at our research centre so that, at sign-up, potential participants can request a female operator to support a study run by a male researcher. The operator is trained in both EEG data collection and Hijabi-friendly procedures and is tasked with fitting the cap and ensuring good signal quality while respecting the participant’s needs.

**Fig. 1:**
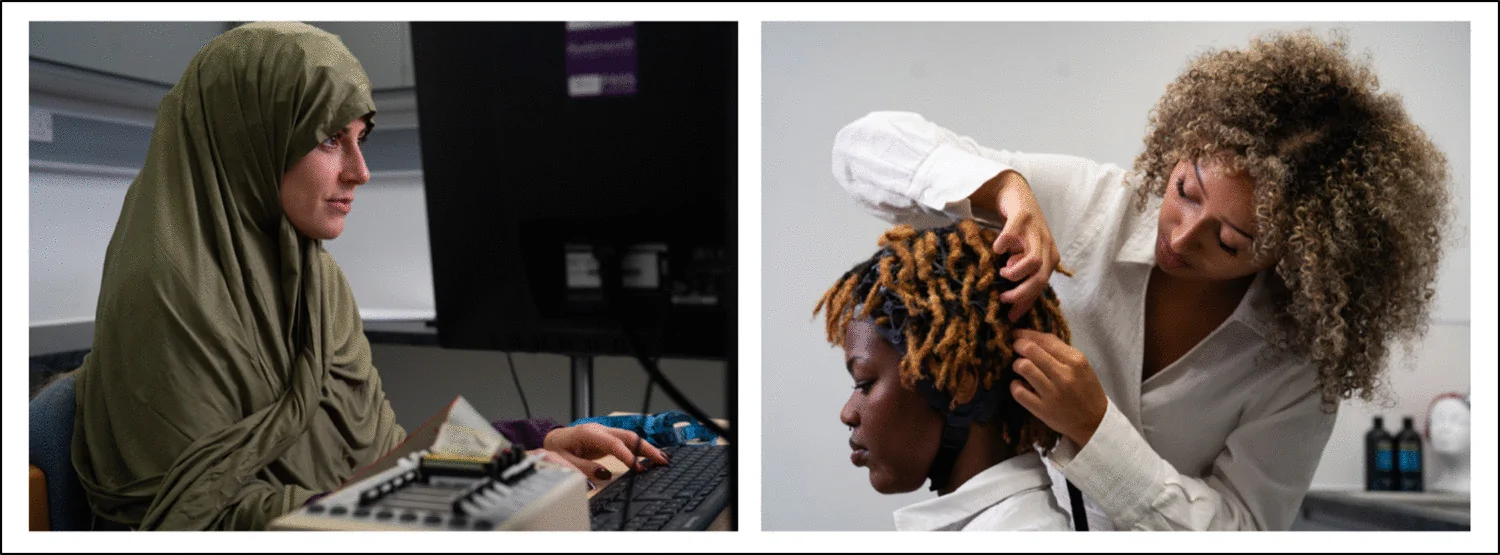
Left panel: A participant wears a hijab on top of an EEG cap (NB: participant is wearing hijab for the purposes of the photo). Right panel: A researcher passes short locs through the gaps in an EEG net to improve electrode contact

We note that male researchers may understandably be wary of the potential for inconsistent delivery of experimental task instructions when working with an operator. Consequently, after the operator fits the EEG cap, the participant can loosely wrap a hijab over the top of the cap to cover any visible hair, thus allowing the male researcher, with consent, to enter the room to discuss the details of the experiment and ensure consistency of the study (Fig. 1). For gel-based systems, the hijabs may get dirty with electrolyte gel, so we recommend having a supply of light-fabric hijabs (e.g., cotton jersey) in the lab, rather than participants dirtying their own. A hijab worn over the EEG cap provides a further sense of security, as removing the hijab is a vulnerable, sacred process for many. Having a supply of lab hijabs is also a clear inclusivity signal to participants, who will then be more likely to return and recommend EEG research in their community. As an additional benefit, a hijab wrapped over the electrode cap provides reassurance for the participant and researcher in the event of unanticipated emergencies, such as building evacuations (see Ricard et al., 2023, for a similar recommendation for magnetic resonance imaging [MRI]).

To help the participant feel secure that they will not be interrupted during their study, clear privacy signage is vital, as well as training for all researchers on appropriate consent to enter the room. Many EEG labs will have a two-room setup—i.e., a control room for the experimenter, and a separate room for the participant. This setup allows for a male researcher to remain in the control room and coordinate the study, while the female operator is with the participant in the separate room. If there is a camera system in the EEG lab, it will need to be switched off or pointed away from the participant. In labs with only one room, a room divider would be required or, preferably, the male researcher would leave until the participant is ready. Signalling to potential participants that researchers understand the privacy requirements of the hijab is also vital, via ‘Hijabi-friendly’ acknowledgement on co-designed recruitment materials, especially in the context of low Muslim representation among researchers.

## EEG procedures: Recommendations by and for Black participants

Many EEG research systems cannot be used effectively with afro-textured hair in its natural state. While innovative technological solutions have been developed (e.g., Etienne et al., 2020; Kwasa et al., 2024), these are typically bespoke devices for afro-textured hair. As EEG researchers would be unlikely to use two separate systems (i.e. one for thinner hair, one for afro-textured hair) within the same experiment due to potential differences in hardware parameters, these approaches are unlikely to provide a clear solution for greater Black inclusivity in non-Black-majority countries. In these cases, we propose that the solutions lie in adapting existing procedures to maximise the potential for Black participation.

From our working group’s experiences, afro-textured hair can be more easily accommodated with net-based EEG systems, as the spaces between electrodes allow the researcher to move hair independently around each electrode and, in some cases, pass braids, twists, and locs through the gaps so that they are not under the electrodes (Fig. 1; see also Hudac et al., 2022), although care is needed to ensure participant comfort during this process (Adams et al., 2024). While net systems are typically saline-based rather than gel-based, it is reassuring that comparable signal quality has been reported between the two electrolytes (Günther et al., 2023). However, as Günther et al. (2023) did not report the ethnicities or hair types of the participants who contributed to that comparison, it is unlikely that a high proportion of them had afro-textured hair (Bradford et al., 2024). Additionally, data suggest that afro-textured hair absorbs water less than Caucasian and Asian hair, which may lead to lower adherence of the saline solution and consequently poorer signal quality (Choy et al., 2022), although these data are based on observations from only one individual (Franbourg et al., 2003). Furthermore, while there is empirical evidence of the impact of hair type on EEG data quality with a gel cap system (Lees et al., 2024), some excellent recommendations exist for improving the applicability of gel cap systems through prior hair preparation (e.g., https://hellobrainlab.com/research/eeg-hair-project/; https://sprweb.org/wp-content/uploads/2025/12/SPR-CPSI-DO-EEG-Hairtypes-Infographic-2025.pdf). Consequently, further empirical research is clearly needed to determine the potential benefits (or otherwise) of saline net systems.

A number of papers and online resources (e.g., Adams et al., 2024, https://hellobrainlab.com/research/eeg-hair-project/) recommend low-profile hairstyles such as a low bun or flat cornrows to maximise the success of the EEG session. Similarly, our working group found that *some* ‘protective hairstyles’ can provide good access to the scalp for conventional EEG. ‘Protective hairstyles’ involve braiding or twisting hair into a style that minimises exposure to heat, dryness, and tangling, thus preventing breakage. In our working group, for example, we have had great success in styling natural hair into cornrows radially from the middle of the head, thus providing good scalp access and a low-profile hairstyle for fitting beneath headcaps (see https://osf.io/x4zd8 for examples, and https://hellobrainlab.com/research/eeg-hair-project/ for additional hairstyling recommendations).

One option, therefore, is to ask the participant to style their hair in advance into a low-profile protective hairstyle, or to have a braider available (similar to the operator above) to style the hair in the lab. However, requiring participants to re-style their hair in advance is a significant additional burden, and some may be uncomfortable with a researcher braiding their hair due to a history of racialised hair discrimination (Mbilishaka et al., 2020). Consequently, clear communication to potential participants that they can participate the next time their hair is in a protective style may be the most inclusive approach. Nevertheless, some electrode locations may still overlap with the locations where the braids meet the scalp. As the braids are immovable at the scalp, these electrodes may need to be moved to locations that deviate from EEG conventions, or they may lack scalp contact entirely and have poor signal quality (Adams et al., 2024). While some ‘bad’ channels are to be expected across all participants regardless of hair phenotype, the field would benefit greatly from empirical evidence of the cost, benefit, and participant tolerance of specific braiding approaches for data quality and consistent electrode placement (Bradford et al., 2024).

Finally, the residue from a gel-based recording will typically require participants to wash their hair after the study. Many labs have hair washing facilities to allow participants to do this before leaving. Therefore, ensuring that hair washing facilities include haircare products for the full range of hair textures is both inclusive and a clear signal to Black participants that EEG research is also for them (Adams et al., 2024).

## Recruitment: Recommendations by and for Hijabis

As our survey data indicate, Hijabi women may be hesitant to sign up for an EEG study because they do not know what accommodations there may be. These feelings may be assuaged in part by making it clear to potential participants that there are processes in place that have been co-created with Hijabis. To that end, we developed a participant-facing video, written and presented by our Hijabi working group, that describes the procedures from a Hijabi perspective, stating explicitly that ‘this video was supported and designed by Hijabi women’. We make this video available through open access (https://www.youtube.com/playlist?list=PLZ0P5qR6sYbY) and encourage its reuse and adaptation for your own EEG contexts (note that videos were not peer-reviewed alongside this article). Similarly, clear descriptions on recruitment materials of Hijabi-friendly procedures (‘Designed by Hijabis, for Hijabis’) signal a welcoming research environment.

## Recruitment: Recommendations by and for Black participants

As discussed above, conventional EEG systems simply have not been designed to work well with afro-textured hairstyles. This, in turn, leads researchers to pre-emptively exclude participants for whom they anticipate data collection challenges. To illustrate this, we reviewed the inclusion/exclusion criteria for all studies advertised on our university’s research participation database that included ‘EEG’ in their titles or descriptions. Of the 76 studies we found, 42 (55%) made explicit reference to hair texture as an exclusion criterion (e.g., ‘No afro/thick hair’). While it is undoubtedly the case that most EEG systems have been designed to work with thinner hair, explicit exclusion of afro-textured hair is problematic because it fails to differentiate between hair texture and hair style. Hairstyles (braids, weaves) are choices that change over time, while textures (afro, kinky) are consistent natural characteristics. Conflating hair type with hairstyle within exclusion criteria signals that afro-textured hair is a problem to be avoided, which is both misleading and alienating (Mbilishaka et al., 2020). Indeed, while some afro-textured hairstyles are likely to be entirely incompatible with conventional EEG (e.g., classic afro, puffs), shorter hairstyles (e.g., a teeny weeny afro) and some protective hairstyles (e.g., cornrows, box braids) may provide the sufficiently low-profile and accessible scalp that an EEG system requires, but would be excluded under less nuanced criteria.

Consequently, we recommend that recruitment adverts and inclusion/exclusion criteria acknowledge limitations respectfully, separate hair texture from hairstyle, use clear language, and invite participants to ask whether their hair will be compatible in its current style. Here we provide co-produced wording you may wish to use and adapt for your own EEG research:

EEG studies involve you wearing an EEG headcap—similar to a swimming-cap lined with a series of electrodes that record the tiny electrical signals generated by your brain. A good recording of EEG data requires the electrodes in this headcap to be close to your scalp. This means that the amount of time taken to set-up the electrodes will vary depending on the amount and style of your hair. You will be reimbursed for this time accordingly. In some rare cases we may be unable to achieve a good recording due to being unable to place the electrodes close enough to your scalp, and so we will need to stop the study. In these cases, you will be reimbursed for the time taken up to that point. If you require a female experimenter to fit the EEG headcap due to your needing to remove your head-covering, please let us know. EEG headcaps require either a conductive gel or saline solution to be applied to your scalp; some participants may wish to wash their hair afterwards. If you wish to do so, we have facilities and products for the full range of hair textures.

As with our Hijabi group, we also co-designed a recruitment video that we make available via open access (https://www.youtube.com/playlist?list=PLZ0P5qR6sYbY) and encourage reuse and adaptation (note that videos were not peer-reviewed alongside this article). This video aims to demystify the EEG procedure and highlight the ways in which EEG research can be Black hair-inclusive. However, to ensure that we do not over-promise in the current technological context, we also make the equipment’s limitations clear and encourage participants to contact our research centre in advance if they have questions about their hairstyle. By reducing exclusions to only those necessitated by the technology, we take a significant step towards greater Black inclusivity in EEG. Ultimately, we achieve our goal of full inclusivity through lobbying of industry for product design that works for all (e.g., https://www.eeg101.eu/).

## Conclusions

Inclusive research practices are a moral imperative and ensure data that accurately reflect and work for all. Overgeneralisation of narrow samples within cognitive EEG risks a research landscape stuck in local minima, while claims about clinical EEG biomarkers carry the greatest moral responsibility for inclusivity (cf. biases in pulse oximetry: Sjoding et al., 2020). We encourage all EEG researchers to actively integrate processes for achieving greater inclusivity at all levels of study design and delivery (see Fox et al., 2024, for an example of good practice). As our survey respondents and working groups emphasised, increasing the diversity of background among EEG researchers is the most effective route to increasing inclusivity of practice. Co-production is a crucial part of this goal, and we encourage all researchers to reach out to under-represented communities. By improving access to EEG research for students, we work to remove the perception that EEG research is only for White people and consequently encourage a new generation of diverse EEG researchers. Towards this end, we also make available an open-access set of photographs of diverse EEG researchers and participants (https://osf.io/x4zd8) that we encourage researchers to use in public-facing and academia-facing communications.

## Data Availability

Raw data and all materials are available to download via https://osf.io/x4zd8.

## Code availability

Data processing and analysis code for the quantitative survey data are available via https://osf.io/x4zd8.

## Declarations

### Open practices statement

Materials are available at https://osf.io/x4zd8.
